# P11 Lubricant-infused silver-polytetrafluoroethylene coated urinary catheters inhibit adhesion of host-secreted proteins and biofilm formation

**DOI:** 10.1093/jacamr/dlad077.015

**Published:** 2023-08-02

**Authors:** Shuai Zhang, Colin P McCoy

**Affiliations:** School of Pharmacy, Queen’s University Belfast, 97 Lisburn Road, BT9 7BL, Belfast, UK; School of Pharmacy, Queen’s University Belfast, 97 Lisburn Road, BT9 7BL, Belfast, UK

## Abstract

**Objectives:**

The adhesion of specific host-derived proteins (particularly fibrinogen) and subsequent biofilm formation play a central role in the pathogenesis of catheter-associated urinary catheter infections (CAUTIs). However, very few catheter materials have proven to be able to simultaneously prevent both bacterial and protein adhesion in a complex physiological environment (i.e. urine). The aims and objectives of this research were to (i) develop silver-polytetrafluoroethylene (AgF)-based coatings with a wide range of surface energies; (ii) test their antifouling performance; and (iii) investigate the influence of surface energy on biofouling accumulation.

**Methods:**

Standard AgF coatings with tailored surface energies (ranging from 18 mJ/m^2^ to 42 mJ/m^2^) were fabricated using an electroless method. By spontaneous polycondensation 1H,1H,2H,2H-perfluorooctyltriethoxysilane (PFOTES) onto the AgF sublayer, a lubricant-infused AgF coating (AgFP) was obtained. The surface morphology, chemical composition, and roughness were characterized by SEM, EDS and AFM, respectively. The surface energy was characterized using a contact angle approach and calculated using the Van Oss method. The anti-adhesion performance of the coated catheters against proteins (fibrinogen [Fgn]; BSA) and bacteria (*Escherichia coli*; *Proteus mirabilis*) was examined and compared with commercial all-silicone catheters.

**Results:**

The results showed that there exist two separate optimum surface energies where bacterial (∼25 mJ/m^2^) and protein adhesion (∼35 mJ/m^2^) are minimal (Figure 1). The deposition of fibrinogen on surfaces significantly accelerated bacterial attachment and biofilm formation, whereas the AgFP coating with the smoothest surface and ultralow surface energy (∼12 mJ/m^2^) displayed significant antibiofilm and anti-protein activities compared with uncoated silicone surface traditional AgF coatings (Figure 2).

**Figure 1.**
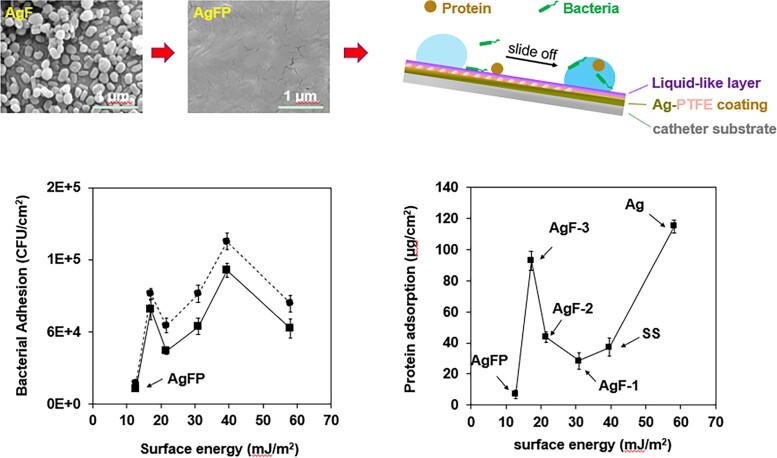
Surface energy on adhesion of bacteria and proteins.

**Figure 2.**
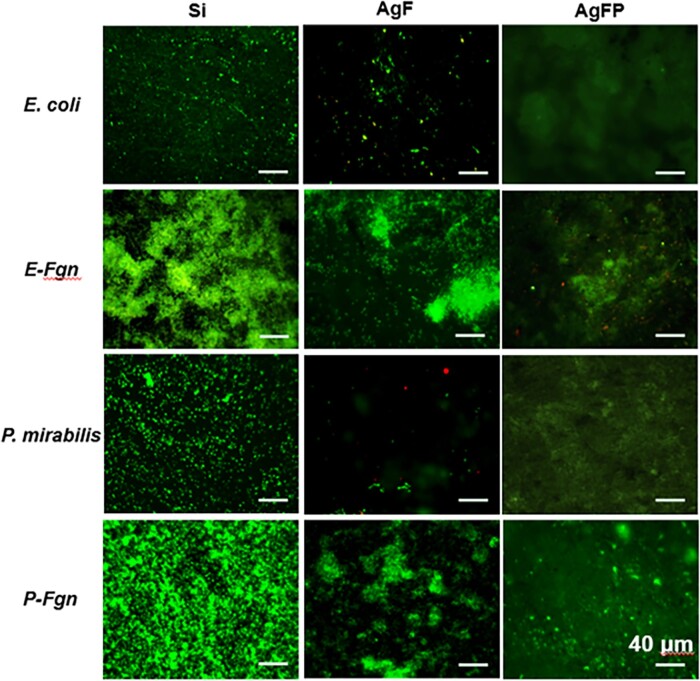
Biofilm formation on Si, AgF and AgFP surfaces after 1 day co-culture in pure bacterial suspension and protein-supplemented bacterial suspension. Scale bars correspond to 40 μm.

**Conclusions:**

The inclusion of lubricant into the AgF matrix leads to the formation of a smooth and super-repelling catheter surface, leading to ultralow adhesion for both bacteria and proteins. Surprisingly, our results indicated that there exists an optimum surface energy where the adhesion of both bacteria and proteins is minimal, which further extended the classic ‘Baier curve’.

